# Residents’ preference trade-offs for critical illness insurance scheme design: a discrete choice experiment in Shandong Province, China

**DOI:** 10.1093/heapol/czag089

**Published:** 2026-07-16

**Authors:** Ruoxuan Zhang, Jingbo Zhang, Huohuo Dai, Xin Zhao, Yibo Wu, Hui Li

**Affiliations:** Department of Otorhinolaryngology, Shandong Provincial Hospital Affiliated to Shandong First Medical University, Jinan, Shandong 250021, China; School of Nursing, Shandong First Medical University, Taian, Shandong 271016, China; Center for Evidence-Based Chinese Medicine, Beijing University of Chinese Medicine, Beijing 100029, China; International Institute of Evidence-Based Traditional Chinese Medicine, Beijing University of Chinese Medicine, Beijing 100029, China; Department of Public Health and Primary Care, Leiden University Medical Center, 2333 ZD Leiden, The Netherlands; National eHealth Living Lab, Leiden University Medical Center, 2333 ZD Leiden, The Netherlands; Department of Otorhinolaryngology, Shandong Provincial Hospital Affiliated to Shandong First Medical University, Jinan, Shandong 250021, China; Department of Nursing, the Fourth Affiliated Hospital of School of Medicine, and International School of Medicine, International Institutes of Medicine, Zhejiang University, Yiwu, Zhejiang 322000, China; Department of Otorhinolaryngology, Shandong Provincial Hospital Affiliated to Shandong First Medical University, Jinan, Shandong 250021, China

**Keywords:** discrete choice experiment, critical illness insurance, scheme design preference

## Abstract

The economic burden of disease remains a significant global public health challenge. In China, the critical illness insurance scheme is a pivotal policy designed to protect households from catastrophic health expenditure. However, evidence on residents’ preferences for this insurance is limited. Identifying these preferences is essential for advancing resident-centered policy reform. To address this gap, we conducted a discrete choice experiment to quantify public preferences for critical illness insurance attributes among residents in Shandong Province, China. Through literature review, qualitative interviews, and expert consultation, we designed a discrete choice experiment with six policy attributes: cost, reimbursement ratio, deductible, annual reimbursement limit, scope of reimbursable expenses, and restriction to designated hospitals. An orthogonal design generated choice sets. Data were collected from 360 residents across 3 cities in Shandong Province and analyzed using mixed logit and latent class models. Latent class analysis identified preference heterogeneity across subgroups. Based on model results, we calculated relative importance scores and simulated selection probabilities. A total of 334 valid questionnaires were collected (46.4% male; 53.6% female). Mixed logit analysis identified the reimbursement ratio (32.73%) as the most important attribute, followed by the annual reimbursement limit (23.81%) and scope of reimbursable expenses (23.10%). Latent class analysis revealed two subgroups: a “Coverage-Intensity Sensitive” Group (Class 1, *n* = 125) and a “Reimbursement-Focused Group” (Class 2, *n* = 209). Class 1 prioritized high reimbursement limits (68.3% relative importance), preferring no annual limit. Class 2 emphasized optimizing the reimbursement ratio (45.6%) and hospital flexibility, while accepting higher deductibles. Policymakers should prioritize increasing reimbursement rates and expanding coverage scope, with appropriate premium adjustments. Aligning critical illness insurance design with residents’ heterogeneous preferences contributes to more targeted and efficient policy arrangement.

Key messages

- China's critical illness insurance scheme operates within a universal health coverage framework, yet evidence on residents’ preferences for this insurance has been limited. Understanding public preferences is essential for designing policies that are both responsive to population needs and fiscally sustainable.
- Using a discrete choice experiment, this study found that residents in Shandong Province prioritize three core benefit attributes: the reimbursement ratio, the annual reimbursement limit, and the scope of coverage for high-cost, nonreimbursable medical services.
- Preferences are not uniform across the population. Latent class analysis identified two distinct subgroups: a “Coverage-Intensity Sensitive” Group that values comprehensive protection and a “Reimbursement-Focused Group” that weighs coverage against cost and prefers flexible options.
- These findings suggest that uniform insurance design cannot fully accommodate residents’ diverse preference demands. Differentiated approaches—such as offering tiered plans with varying combinations of reimbursement rates, coverage ceilings, and deductible levels—could better align with heterogeneous public preferences and enhance financial protection.

## Introduction

The economic burden of disease is a persistent challenge for healthcare systems worldwide (Global Burden of Disease Health Financing Collaborator Network 2017, 2018). With rapid economic development and rising living standards, public demand for comprehensive health security has risen substantially (GBD 2019 Human Resources for Health Collaborators 2022). However, the high prevalence of critical illnesses and high medical costs continue to impose severe financial burdens on many households, and poverty due to illness remains a concern (Liu et al. 2007, Wagstaff et al. 2018).

To address this challenge, the Chinese government launched the critical illness insurance (CII) scheme for residents in 2012, as a key component of efforts to strengthen financial protection and improve equity in healthcare (Central People's Government of the People's Republic of China 2026). The scheme has been fully implemented since its introduction (National Healthcare Security Administration 2026). It functions as a supplement to basic medical insurance by covering a portion of out-of-pocket costs for catastrophic illnesses, thereby extending financial protection and reducing the economic impact on patients with severe diseases. Evidence suggests that CII policies help reduce catastrophic medical expenditures (Fu 2022, Li et al. 2025).

In China, the basic health insurance system consists of two main schemes: the Urban Employee Basic Medical Insurance (UEBMI) for formal sector workers and the Urban–Rural Resident Basic Medical Insurance (URRBMI) for self-employed individuals, rural residents, the elderly, and children (Zhou et al. 2021). The CII scheme operates as an automatic, supplementary component integrated into both basic medical insurance programs, with no voluntary opt-in or separate enrollment process (Central People's Government of the People's Republic of China 2026). It provides additional reimbursement for high medical expenses that exceed the coverage cap of basic insurance, primarily targeting catastrophic health expenditure. Current CII policies mainly cover expensive treatments for severe diseases included in the official list of critical illnesses, while certain high-cost services, specialized drugs, and nondesignated medical services may remain outside the scope of reimbursement.

Existing literature has documented the critical role of health insurance in mitigating catastrophic health expenditure and reducing medical impoverishment in other developing economies. For example, studies in Vietnam have illustrated how health financing arrangements affect the incidence of catastrophic and impoverishing health care payments (Wagstaff and Van Doorslaer 2003). Research in India has further examined the importance of health insurance for financial protection against high-cost illnesses (Joglekar 2008) and identified key socioeconomic determinants that influence insurance uptake and coverage penetration among different population groups (Chakrabarti and Shankar 2015). Despite this growing international evidence, few studies have systematically explored residents’ preferences for the specific benefit attributes of CII in the Chinese policy context.

Most existing studies focus on evaluating policy outcomes (Duan et al. 2025, Titaley et al. 2025), while less attention has been paid to residents’ preferences for specific insurance attributes, such as reimbursement ratios, deductibles, and reimbursement limits. This study aims to address this gap.

As a populous and economically diverse province in China, Shandong Province offers a suitable context to examine how residents with different socioeconomic backgrounds perceive and value CII schemes. The province was also among the first to implement the CII Framework in 2013 (Shandong Provincial People's Government 2025). Longitudinal evaluations show that the policy has achieved positive outcomes: broader basic medical insurance coverage, upgraded benefit packages, and reduced household financial burden associated with medical spending (Jiang et al. 2019, Zhai et al. 2021). However, socioeconomic changes and rising public expectations have also revealed challenges, such as uneven public acceptance and varying levels of policy understanding among beneficiaries (Ding et al. 2025).

To understand residents’ scheme design preference, a method is needed that can capture their trade-offs among multiple policy attributes. This study employed a discrete choice experiment (DCE) to quantitatively analyze the residents’ scheme design preferences in Shandong Province (Clark et al. 2014). Grounded in random utility theory, DCEs present respondents with experimentally designed scenarios and choice sets to simulate real-world decisions. This approach enables the identification of latent preferences. The DCE method has been widely applied in health economics, as well as in research on health behaviors and healthcare utilization (Soekhai et al. 2019, Xiao et al. 2023, Zhang et al. 2023). However, its application to exploring scheme design preferences for China's CII has been limited. Applying the DCE in this context can be used to quantify residents’ preferences by determining the relative importance they assign to key policy attributes. This application addresses a research gap and can provide evidence to inform policy optimization.

## Materials and methods

### Study design

This study employed a DCE within a cross-sectional survey conducted among residents of Shandong Province (Clark et al. 2014). In the DCE framework, participants were presented with pairs of hypothetical insurance schemes. Each scheme was defined by specific combinations of attributes and levels, and participants were asked to choose the scheme they preferred in each pair.

### Identification of discrete choice experiment attributes and level

The development of attributes and levels followed established guidelines for DCEs (Bridges et al. 2011). A combination of qualitative and quantitative approaches was used. These were identified and refined through a review of previous DCE studies on health insurance, expert consultations, and interviews with potential participants (Gething et al. 2024).

#### Literature review

A systematic literature review was conducted to identify key attributes and levels used in previous DCE studies on health insurance and CII schemes. Six electronic databases were searched, including PubMed, Web of Science, Embase, Scopus, China National Knowledge Infrastructure (CNKI), and Wanfang, covering both international and Chinese studies (Abiiro et al. 2014, Kazemi Karyani et al. 2019, Yan et al. 2022, Ma et al. 2023, Rahmani et al. 2023, Chen et al. 2025). Relevant attributes related to benefit design, cost, reimbursement policies, and coverage scope were extracted and compiled into an initial candidate pool. This step ensured alignment with existing academic evidence and established preference attributes in health insurance research.

#### Expert consultation

Four experts in public health, health security policy, health management, and health services research were consulted via semi-structured online interviews in November and December 2024. Experts were asked to evaluate the relevance, clarity, feasibility, and policy consistency of candidate attributes and levels. Their feedback was used to remove redundant items, refine wording, adjust level ranges, and ensure alignment with current Shandong Province and national CII policy regulations.

#### Resident interviews

Preliminary qualitative interviews were conducted with potential participants to confirm understanding, appropriateness, and real-world relevance of the attributes. Residents commented on the practical importance of each policy feature, the reasonableness of level ranges, and the clarity of scenario descriptions. Input from residents was used to further simplify expression, optimize level intervals, and ensure that the choice tasks reflected realistic decision-making contexts.

Based on the above three steps, six core attributes were retained to avoid respondent burden while ensuring key trade-offs could be captured. Each attribute was assigned two to four levels consistent with actual policy practice. The final attributes and levels are presented in Table 1.

**Table 1:** Attributes and levels for DCE choice questions.

Attributes	Levels	Definition
Cost	¥440	Annual premium for the CII scheme.
	¥470	
	¥500	
	¥530	
Reimbursement ratio	60%	The percentage of eligible medical expenses reimbursed by the scheme.
	65%	
	70%	
	75%	
Deductible	¥10 000	The amount of eligible medical expenses an individual must pay out-of-pocket before the insurance begins reimbursement.
	¥15 000	
	¥20 000	
Annual reimbursement limit	¥400 000	The maximum total amount the scheme will reimburse in a given year.
	¥500 000	
	¥600 000	
	boundless	
Expansion of reimbursement coverage	Special medical services in the top 10% of out-of-catalog expenses	The scope of covered medical services beyond the basic insurance package.
	Special medical services in the top 20% of out-of-catalog expenses	
	Special medical services in the top 30% of out-of-catalog expenses	
Designated hospitals	Yes	Whether reimbursement is restricted to services received at hospitals contracted with the insurance scheme.
	No	

The premium levels were based on the current cost of basic medical insurance for residents in Shandong Province. The lowest level (¥30) approximated the typical annual increase in the basic insurance premium. The highest level (¥530) was set ∼20%–30% above the current premium to test willingness to pay. The levels for the reimbursement ratio, deductible, and annual reimbursement limit were informed by current CII policies in several provinces and cities, reflecting actual policy variations and feasibility. For expanding the scope of reimbursable expenses, the focus was on high-cost special medical services not currently covered. Potential services were ranked by cost, and scaled additions (e.g. increments representing ∼10% of high-cost services) were used to balance resident needs with fund sustainability, avoiding extreme values. The inclusion of an attribute on designated hospitals (i.e. whether reimbursement is restricted to contracted providers) was designed to assess the value residents place on freedom of choice in healthcare access.

### Questionnaire design and quality control

**Figure 1:**
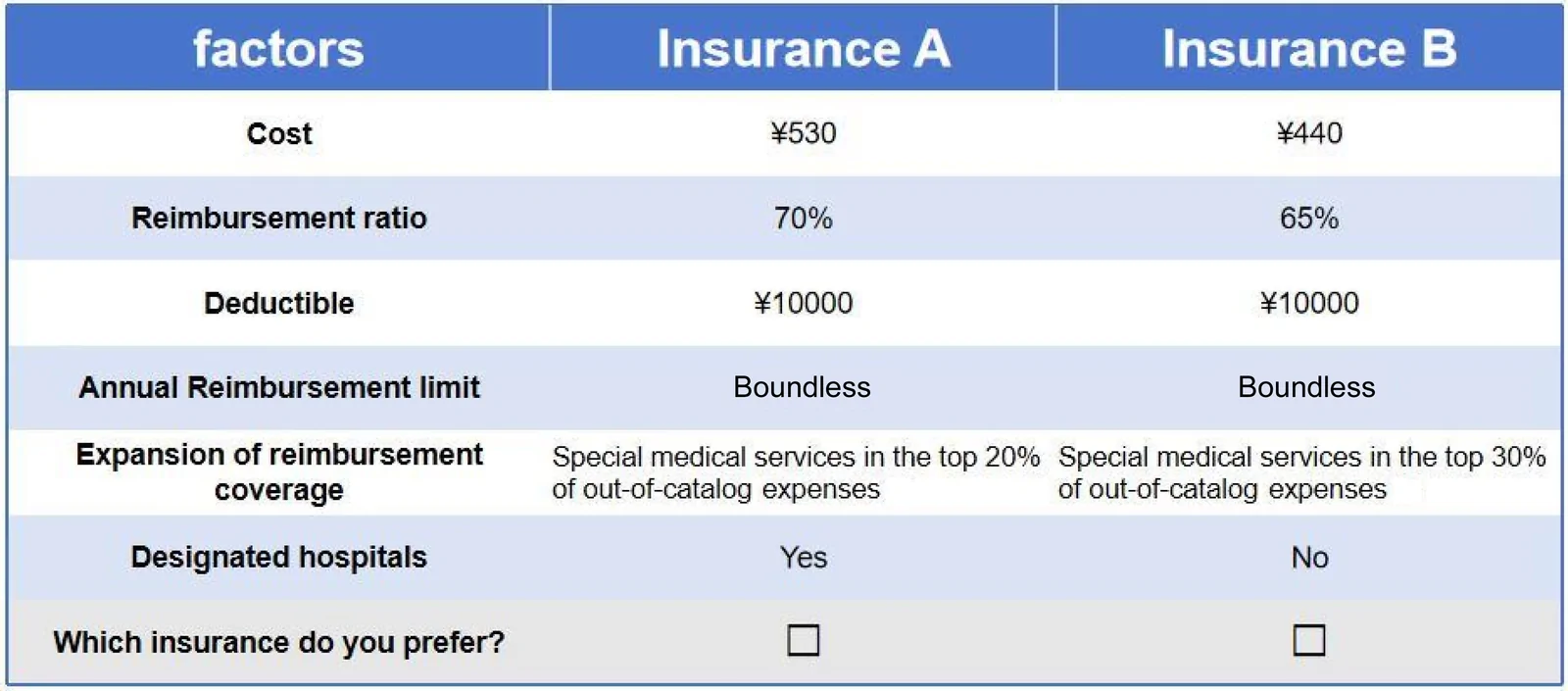
An example of a discrete choice question presented to participants.

The questionnaire consisted of three sections: (i) study introduction and objectives, (ii) participants’ sociodemographic characteristics, and (iii) DCE tasks. The DCE was based on six attributes, each with two to four levels. An orthogonal main-effects design was used to manage the complexity of evaluating all possible attribute-level combinations and to minimize correlation among choice sets. The design generated 24 distinct choice sets for the survey. Each choice set presented 2 alternative insurance schemes, and each respondent was assigned a block of 12 choice sets. An opt-out option was not included in the choice sets. A sample choice task is presented in Fig. 1 .

### Study population

The minimum sample size was estimated using Orme’s equation, a widely validated method for discrete choice experiments (de Bekker-Grob et al. 2015); a minimum sample size of 84 was required.

Participants were required to meet the following inclusion criteria: (i) aged 18 years and above, (ii) holding Chinese citizenship, (iii) residing in Shandong Province, and (iv) being able to finish the questionnaire independently or with the investigator’s assistance when necessary. Individuals were excluded according to the following standards: (i) those diagnosed with cognitive impairment or psychiatric disorders and (ii) those who declined to participate after receiving a detailed explanation of the study.

A multistage sampling approach was used. First, Shandong Province was selected as the study site for three key reasons: (i) it is an economically and demographically representative province in eastern China, with diverse urban–rural development patterns; (ii) Shandong was among the earliest provinces to implement the CII policy in 2013, providing a mature policy environment; and (iii) it has a large resident population, allowing sufficient sample heterogeneity for a DCE study.

Within Shandong, three cities (Jinan, Tai′an, and Weifang) were purposively selected to represent regional socioeconomic variation: Jinan as the provincial capital with relatively high economic development, Weifang as a typical mid-level city, and Tai′an as a relatively less developed inland city. This stratification helped ensure the sample reflected diverse resident preferences across varying economic and policy implementation contexts.

Within each selected city, two to three communities were randomly chosen from official listings. A minimum of 30 questionnaires were collected from each community. Although random selection within cities strengthens internal validity, this approach may not yield a sample that is fully representative of the provincial population. This is an acknowledged limitation in early-stage DCE studies.

Between February and April 2025, a survey on scheme design preferences for CII was conducted among residents of the ten selected communities. To ensure data quality, the questionnaire included a duplicate choice task as an internal consistency check. Two trained researchers administered the surveys. They received standardized training to follow a consistent protocol, ensuring that respondents read and understood the choice tasks before proceeding. The average time to complete the questionnaire was ∼20 minutes.

### Data analysis

Descriptive statistics were used to summarize the sociodemographic characteristics of participants. The DCE data were analyzed using multiple models, including conditional logit (CL), mixed logit (MXL), and latent class logit (LCLM) models. Model fit was compared using the Akaike Information Criterion (AIC) and Bayesian Information Criterion (BIC). Lower AIC and BIC values indicate better model fit (Vrieze 2012).

The CL model estimates mean preference coefficients for the overall sample. However, it does not capture unobserved preference heterogeneity across individuals (Prosser 2016). To address this limitation, we first used a MXL model, which is specifically designed to capture unobserved preference heterogeneity. We then applied latent class analysis (LCA) to identify distinct preference subgroups. LCA assumes that preferences vary systematically across latent subgroups within the sample. The model estimates both the probability of an individual belonging to each class and the class-specific preference parameters. The relative importance score measures the contribution of each attribute to the overall choice. It is calculated as the part-worth utility range for that attribute divided by the sum of the utility ranges across all attributes.

Simulated choice probabilities for different insurance schemes were calculated using the estimated model coefficients. These simulations quantify how changes in attribute levels, relative to a baseline scheme, affect the predicted preference for a given insurance plan.

Predicted choice probabilities were estimated using the CL model, which provides population-averaged preference coefficients for scenario comparisons. Probabilities were calculated using the estimated utility coefficients according to the standard logit formula: the probability of choosing scheme *j* equals the exponential value of the systematic utility of scheme *j*, divided by the sum of the exponential values of utility for all schemes within the choice set. A baseline scheme was constructed using the least preferred level for each attribute, based on the estimated preference coefficients. Counterfactual simulations were then conducted by improving one or more attributes while holding all other levels constant at the baseline. Uncertainty was not explicitly quantified in the simulation, as the analysis focused on relative changes in choice probability under alternative policy designs. Given the forced-choice experimental design without an opt-out alternative, predicted probabilities reflect relative preference strength among hypothetical insurance schemes, rather than actual enrollment or uptake rates in a real-world setting.

All statistical analyses were conducted using Stata 17.0 (StataCorp LP, College Station, TX, USA). Statistical significance was defined at *α* = 0.05 (two-tailed).

## Results

### Descriptive statistics

A total of 360 respondents completed the survey. After quality checks, 334 valid responses were retained, resulting in a response rate of 92.8%. The majority of participants (76.9%) were under 45 years of age, and 53.6% were female. Most respondents (76.9%) were married, and 51.2% had an educational attainment of junior college or below. Currently employed individuals made up 65.3% of the sample. The demographic structure of the study sample differed from the general population of Shandong Province, with an overrepresentation of individuals under 45 years old and middle-to-high income groups. This sampling structure may limit the representativeness of the findings for older, retired, or low-income populations. Table 2 presents the complete sociodemographic characteristics of the sample.

**Table 2:** Sociodemographic characteristics of the total sample and latent classes.

Characteristics	Full sample	Coverage-Intensity Sensitive group	Cost-benefit balancing group
Sample size	334 (100.0)	125 (37.4)	209 (62.6)
Age (years)			
18–25	55 (16.5)	15 (12.0)	40 (19.1)
25–35	80 (24.0)	25 (20.0)	55 (26.3)
35–45	122 (36.5)	56 (44.8)	66 (31.6)
45–55	58 (17.4)	21 (16.8)	37 (17.7)
>55	19 (5.7)	8 (6.4)	11 (5.3)
Gender			
Male	155 (46.4)	62 (49.6)	93 (44.5)
Female	179 (53.6)	63 (50.4)	116 (55.5)
Household registration system			
Rural household registration	162 (48.5)	68 (54.4)	94 (45.0)
Urban household registration	172 (51.5)	57 (45.6)	115 (55.0)
Marriage status			
Never married	72 (21.6)	19 (15.2)	53 (25.4)
Married	257 (76.9)	104 (83.5)	153 (73.2)
Widowed	1 (0.3)	0	1 (0.5)
Divorced	4 (1.2)	2 (1.6)	2 (1.0)
Number of children			
0	85 (25.4)	25 (20.8)	59 (28.2)
1	116 (34.7)	52 (41.6)	64 (30.6)
2	121 (36.2)	42 (33.6)	79 (37.8)
≥3	12 (3.6)	5 (4.0)	7 (3.3)
Education attainment			
Junior secondary education or below	44 (13.2)	18 (14.4)	26 (12.4)
Vocational secondary education	21 (6.3)	6 (4.8)	15 (7.2)
Senior secondary education	35 (10.5)	21 (16.8)	14 (6.7)
Associate degree	71 (21.3)	29 (23.2)	42 (20.1)
Bachelor's degree	132 (39.5)	41 (32.8)	91 (43.5)
Master's degree or above	31 (9.3)	10 (8.0)	21 (10.0)
Employment status			
Employed	218 (65.3)	92 (73.6)	126 (60.3)
Student	27 (8.1)	6 (4.8)	21 (10.0)
Retired	11 (3.3)	1 (0.8)	10 (4.8)
Self-employed/informal sector worker	66 (19.8)	20 (16.0)	46 (22.0)
Unemployed	2 (0.6)	1 (0.8)	1 (0.5)
Not in the labor force	10 (3.0)	5 (4.0)	5 (2.4)
Monthly income (Renminbi, RMB)			
≤2000	44 (13.2)	12 (9.6)	32 (15.3)
2001–4000	55 (16.5)	26 (20.8)	29 (13.9)
4001–6000	86 (25.7)	39 (31.2)	47 (22.5)
6001–8000	46 (13.8)	11 (8.8)	35 (16.7)
8001–10 000	54 (16.2)	18 (14.4)	36 (17.2)
≥10 001	49 (14.7)	19 (15.2)	30 (14.4)
Health insurance			
Government-paid medical scheme	19 (5.7)	12 (9.6)	7 (3.3)
Urban Resident Basic Medical Insurance	105 (31.4)	42 (33.6)	63 (30.1)
Urban Employee Basic Medical Insurance	133 (39.8)	42 (33.6)	91 (43.5)
New Rural Cooperative Medical Scheme	59 (17.7)	25 (20.0)	34 (16.3)
Commercial health insurance	13 (3.9)	3 (2.4)	10 (4.8)
Uninsured	5 (1.5)	1 (0.8)	4 (1.9)

### Model estimation of preferences

Regression analysis was performed using both CL and MXL models. The AIC and BIC were calculated for each model to assess model fit. As shown in Table 3 and Supplementary Table S1, the MXL model had lower AIC and BIC values (AIC = 4771.054; BIC = 4931.8) than the CL model (AIC = 4871.06; BIC = 4954.927). These lower values indicate that the MXL model provided a better fit to the data. Consequently, the DCE findings are primarily interpreted based on the MXL model estimates.

**Table 3:** Estimates of the MXL model.

Attribute	*β*	Standard error (SE)	*P*	95% CI
Cost	0.001	0.003	.710	(−0.004 to 0.006)
Reimbursement ratio (reference 60%)				
65%	−0.778	0.286	.007	(−1.339 to −0.216)
70%	0.782	0.253	.002	(0.287–1.278)
75%	1.586	0.154	.000	(1.285–1.888)
Deductible (reference ¥10 000)				
¥15 000	−0.305	0.213	.152	(−0.722 to 0.112)
¥20 000	−1.149	0.656	.080	(−2.434 to 0.136)
Annual reimbursement limit (reference ¥400 000)				
¥500 000	−0.453	0.203	.026	(−0.850 to −0.055)
¥600 000	1.182	0.367	.001	(0.463–1.901)
Boundless	1.679	0.495	.001	(0.709–2.648)
Expansion of reimbursement coverage (reference special medical services in the top 10% of out-of-catalog expenses)				
Special medical services in the top 20% of out-of-catalog expenses	0.789	0.170	.000	(0.456–1.121)
Special medical services in the top 30% of out-of-catalog expenses	1.584	0.270	.000	(1.055–2.114)
Designated hospitals (reference yes)				
No	0.215	0.338	.525	(−0.448 to 0.878)
Log-likelihood	−2362.9083			
AIC	4771.817			
BIC	4932.568			

**Figure 2:**
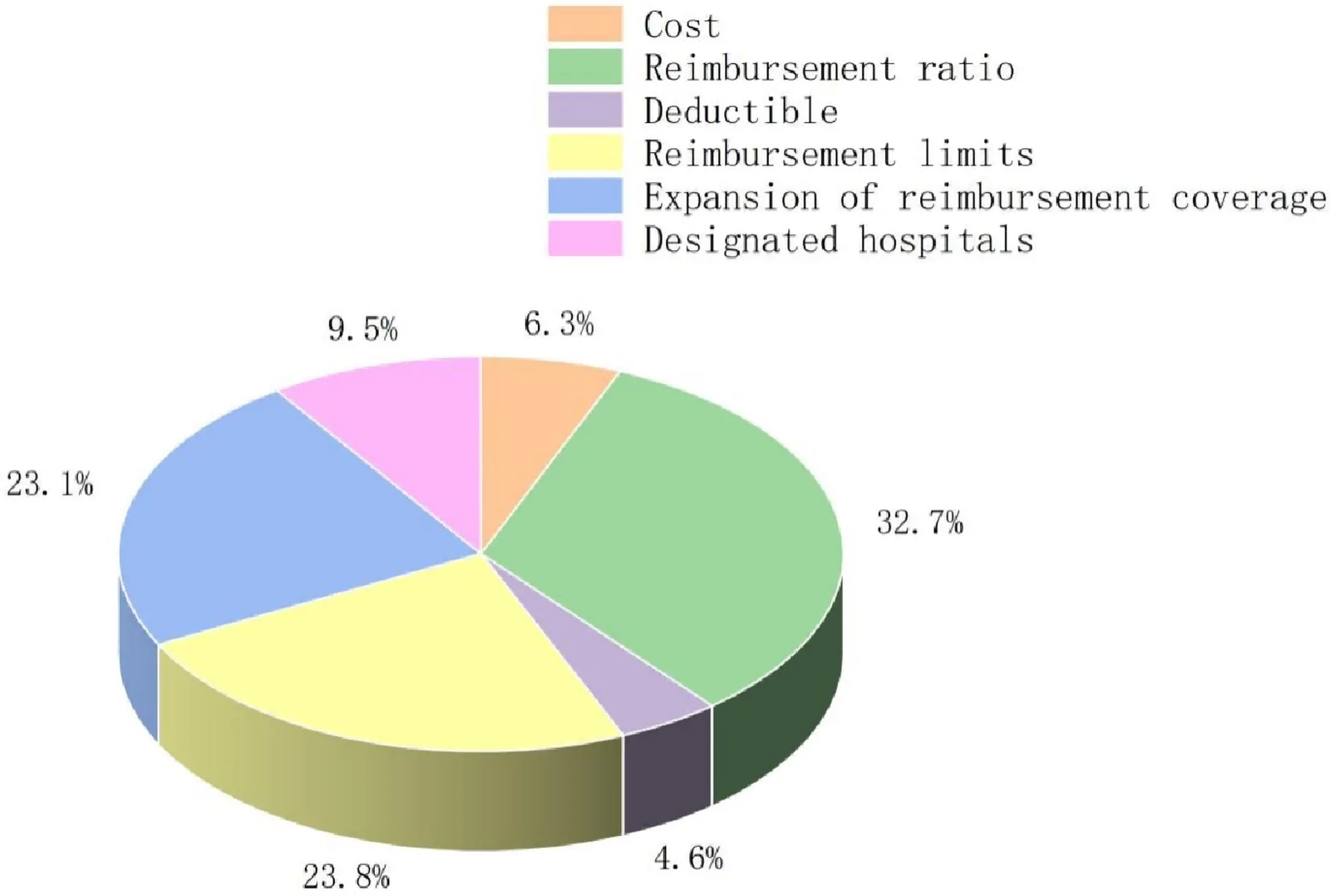
The relative importance of each attribute.

In Table 3, the regression results indicated that cost, deductible, and the requirement for designated hospitals were not significant. In contrast, reimbursement ratio, reimbursement limit, and the expansion of coverage significantly shaped residents’ preferences for CII scheme design. When using a 60% reimbursement ratio as the reference, a 65% ratio was associated with a negative coefficient [*β* = −0.780, 95% confidence interval (CI): −1.341 to −0.219]. Ratios of 70% and 75% had positive coefficients (*β* = 0.778, 95% CI: 0.283–1.274; *β* = 1.586, 95% CI: 1.285–1.888, respectively).Compared with a ¥400 000 annual reimbursement limit, a limit of ¥500 000 had a negative coefficient (*β* = −0.452, 95% CI: −0.85 to −0.055). Higher limits of ¥600 000 and unlimited coverage had significant positive coefficients (*β* = 1.183, 95% CI: 0.464–1.902; *β* = 1.678, 95% CI: 0.708–2.647). Expanding the coverage scope from the top 10% to the top 20% and 30% of high-cost, nonreimbursable services was associated with progressively higher positive coefficients (*β* = 0.788, 95% CI: 0.455–1.12; *β* = 1.582, 95% CI: 1.053–2.112). This pattern suggests a preference for more comprehensive coverage of high-cost therapies. The relative importance of each attribute is shown in Fig. 2.

### Latent class logit model

The latent class logit model (LCLM) analysis identified heterogeneity in preferences for CII. To identify preference subgroups, latent class logit models with one, two, and three classes were estimated. Model fit was assessed using the log-likelihood, AIC, BIC, and entropy. The three-class model failed to converge reliably and produced very small class sizes, limiting substantive interpretation. The two-class model was therefore selected as the preferred solution, as it provided stable estimates, clear class separation, and meaningful preference patterns. As shown in Table 4, the analysis identified two latent classes with distinct preference patterns: Class 1 (Coverage-Intensity Sensitive Group) and Class 2 (Reimbursement-Focused Group).

**Table 4:** Estimates of the LCLM.

Attribute	Coverage-Intensity Sensitive Group				Reimbursement-Focused Group			
	*β*	SE	*P*	95% CI	*β*	SE	*P*	95% CI
Cost	−0.001	0.005	.819	(−0.010 to 0.008)	0.010	0.006	.059	(−0.002 to 0.023)
Reimbursement ratio (reference 60%)								
65%	−1.830	0.406	.000	(−2.626 to 1.036)	0.205	0.333	.650	(−0.447 to 0.857)
70%	−0.120	0.456	.731	(−1.015 to 0.774)	2.147	1.035	.007	(0.118–4.176)
75%	0.844	0.128	.000	(0.594–1.094)	0.971	0.133	.000	(0.710–1.231)
Deductible (reference ¥10 000)								
¥15 000	−0.137	0.322	.717	(−0.769 to 0.495)	0.703	0.657	.263	(−0.586 to 1.991)
¥20 000	−2.844	0.524	.023	(−3.871 to 1.817)	2.244	1.156	.129	(−0.022 to 4.509)
Annual reimbursement limit (reference ¥400 000)								
¥500 000	0.202	0.312	.590	(−0.411 to 0.814)	−1.132	0.522	.005	(−2.156 to 0.109)
¥600 000	2.394	0.349	.001	(1.710–3.078)	−0.391	0.326	.518	(−1.029 to 0.248)
Boundless	2.576	0.468	.007	(1.658–3.494)	−0.101	0.433	.893	(−0.950 to 0.748)
Expansion of reimbursement coverage (reference special medical services in the top 10% of out-of-catalog expenses)								
Reference special medical services in the top 20% of out-of-catalog expenses	0.557	0.250	.090	(0.067–1.047)	1.289	0.718	.003	(−0.119 to 2.696)
Reference special medical services in the top 30% of out-of-catalog expenses	0.994	0.263	.016	(0.478–1.510)	1.918	0.728	.000	(0.490–3.345)
Designated hospitals (reference yes)								
No	−1.761	0.295	.010	(−2.338 to 1.184)	2.296	0.703	.004	(0.918–3.674)

Within Class 1 (Coverage-Intensity Sensitive Group), the negative coefficient for the 65% reimbursement rate (*β* = −1.830, 95% CI: −2.626 to −1.036) indicates an aversion to lower coverage levels. Conversely, the positive coefficient for the 75% reimbursement level (*β* = 0.844, 95% CI: 0.594–1.094) suggests a preference for higher financial protection. This group also showed a strong positive utility for the ¥600 000 annual reimbursement limit (*β* = 2.394, 95% CI: 1.710–3.078). However, it had substantial negative coefficients for increased deductibles (e.g. ¥20 000 deductible: *β* = −2.844, 95% CI: −3.871 to −1.817). This pattern reflects an orientation toward comprehensive coverage and a strong aversion to high out-of-pocket costs.

Within Class 2 (Reimbursement-Focused Group), preferences were primarily driven by the reimbursement ratio and coverage scope. The significant positive coefficient for the 70% reimbursement rate (*β* = 2.147, 95% CI: 0.118–4.176, *P* = .007) indicates a strong preference for improved reimbursement levels. In contrast, the coefficient for the ¥500 000 annual reimbursement limit was significantly negative (*β* = −1.132, 95% CI: −2.156 to −0.109, *P* = .005), suggesting a lower preference for moderate reimbursement limits relative to the reference level. Coefficients for premium and deductible were not statistically significant (*P* > .05), so no firm conclusions about cost or cost-sharing sensitivity can be drawn for this class.

A comparison of the demographic characteristics between the two groups demonstrated that there are distinct subgroups with different preferences. The Coverage-Intensity Sensitive Group (Class 1) tended to be older, more likely to be married, and employed. Their preference pattern placed higher value on extensive and strong coverage. In contrast, the Reimbursement-Focused Group (Class 2) tended to be younger and had more diverse employment statuses. Their preferences were centered on maximizing reimbursement-related benefits, including higher reimbursement ratios and expanded coverage scopes. The sociodemographic profiles of these latent classes are presented in Table 2.

### Predicting choice probability

**Figure 3:**
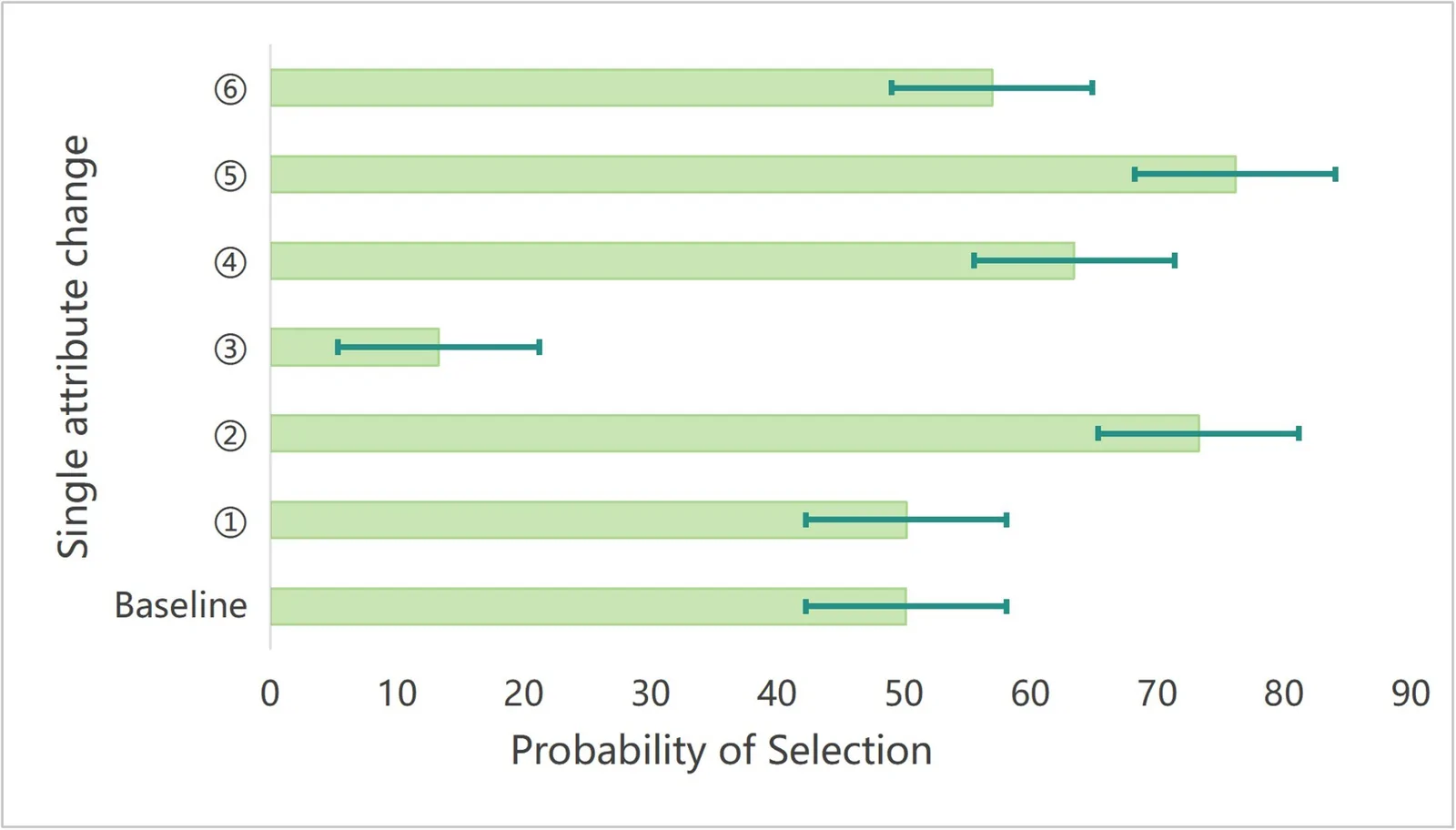
Predicted probability of selection under single-attribute-change scenarios. Note: Baseline is defined as all other attributes being at the reference level (premium of ¥440, reimbursement rate of 60%, deductible of ¥20 000, annual reimbursement limit of ¥400 000, expanded coverage scope including the top 10% of out-of-directory medical items, and restricted to designated hospitals); ① = premium ¥530; ② = reimbursement rate of 75%; ③ = deductible of ¥10 000; ④ = unlimited reimbursement limit; ⑤ = expanded scope covering the top 30% of out-of-directory medical items; ⑥ = nondesignated hospitals included.

**Figure 4:**
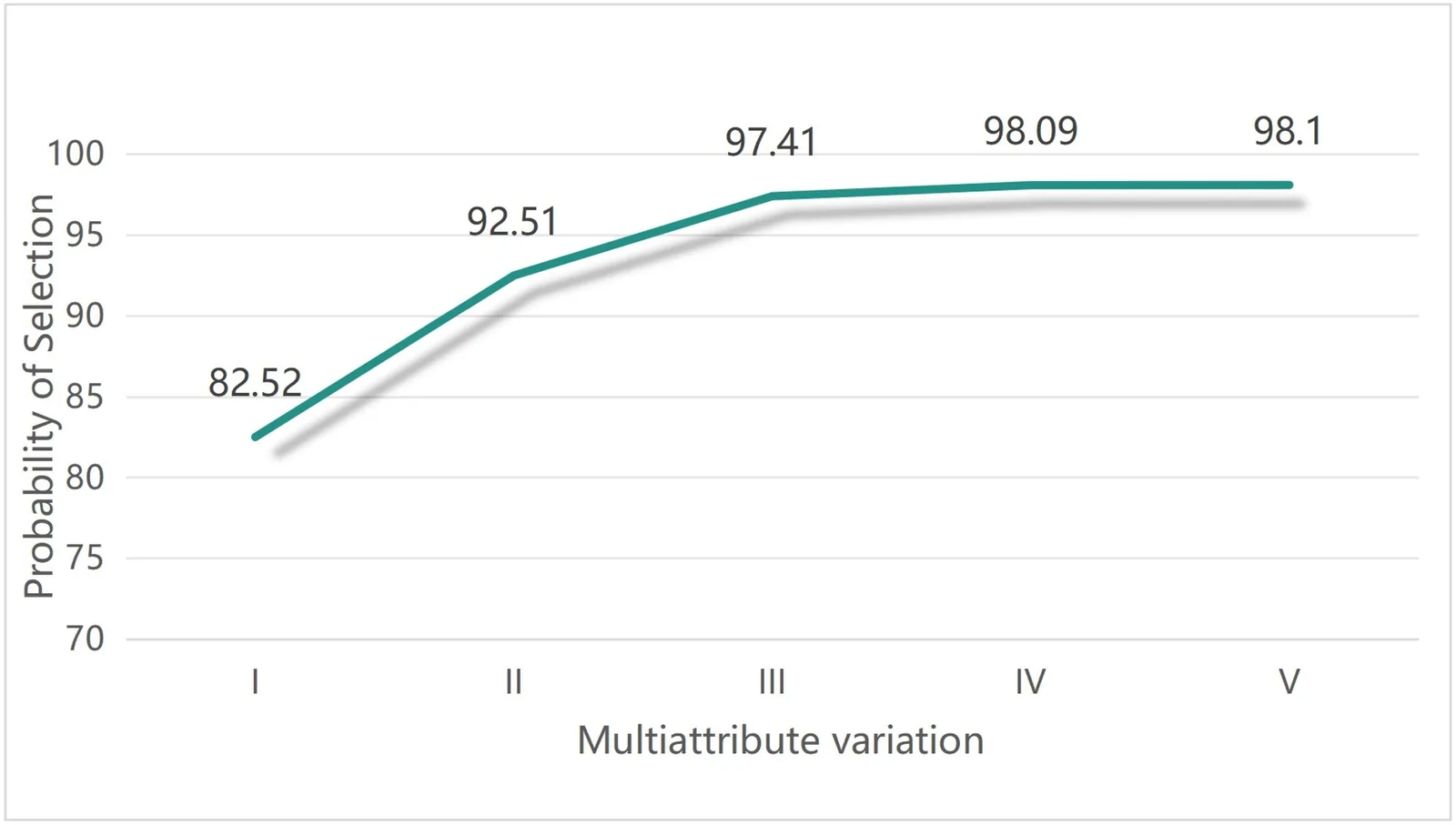
Predicted probability of selection under multiattribute-change scenarios. Note: Each scenario (I–V) represents a cumulative improvement from the baseline defined in Fig. 3. The added attribute changes for each scenario are listed below: Scenario I: Reimbursement rate increased to 75%; deductible reduced to ¥10 000. Scenario II: Scenario I plus an unlimited annual reimbursement limit. Scenario III: Scenario II plus the coverage scope expanded to the top 30% of high-cost, nonreimbursable medical items. Scenario IV: Scenario III plus hospital access unrestricted (nondesignated hospitals included). Scenario V: Scenario IV plus the premium increased to ¥530.

As shown in Figs 3 and 4, the MXL model was used to simulate changes in choice probabilities for different insurance plans under various scenarios. The baseline scenario had the following characteristics:

- Premium: ¥440.
- Reimbursement rate: 60%.
- Deductible: ¥20 000.
- Annual reimbursement limit: ¥400 000.
- Coverage scope: top 10% of high-cost nonreimbursable items.
- Designated hospitals: required.

The predicted selection probability for this baseline plan was 50.16%. This baseline probability reflects the forced-choice design of the experiment, which did not include an opt-out option.

When a single attribute was changed from its baseline level, expanding the coverage scope had the largest positive effect on the choice probability. When multiple attributes were simultaneously improved, the choice probability increased progressively. The highest predicted choice probability was for a plan combining the most favorable level of each attribute: a premium of ¥530, a 75% reimbursement rate, a ¥10 000 deductible, no annual reimbursement limit, coverage of the top 30% of high-cost items, and no restriction to designated hospitals.

## Discussion

This study used a DCE with a sample of residents from Shandong Province, China, to examine scheme design preferences for CII. As shown in the MXL results (Table 3) and relative importance scores (Fig. 2), the analysis identified three core benefit attributes—reimbursement ratio, reimbursement limit, and expansion of coverage—that significantly influenced the choice of insurance schemes (*P* < .05). Because the sample was relatively young and included a high share of middle-to-high income respondents, preferences among elderly or retired groups—who face greater risks of critical illness—may differ from those estimated in this study.

### Core preference attributes and resource prioritization trade-offs

As illustrated in Fig. 2, the reimbursement ratio had the highest relative importance among the attributes studied. Empirical evidence indicates that a higher reimbursement ratio can effectively reduce the incidence of catastrophic health expenditure for lower-middle income households (Gruber and Levy 2009). Consistent with previous studies (Einav et al. 2010, Xie and Hu 2022), a higher reimbursement ratio was associated with a greater preference for an insurance scheme. Given the high treatment costs associated with critical illnesses (Richard et al. 2018, Odahowski et al. 2019), this observed preference for higher reimbursement rates aligns with risk aversion theory (Kahneman and Tversky 1979), as individuals seek to minimize potential financial losses from uncertain, high medical costs. However, existing research suggests that excessively high reimbursement rates may introduce moral hazard and adverse selection, potentially affecting insurance fund sustainability (Einav et al. 2010). Therefore, policy design requires balancing high reimbursement levels with mechanisms to mitigate potential market imbalances and ensure long-term sustainability.

Consistent with Table 3 and Fig. 2, the reimbursement limit was the second most important attribute. This observed preference may reflect concerns related to the risk of extreme medical costs. Catastrophic illnesses, such as certain cancers requiring annual treatment costs of ¥200 000–¥500 000, can lead to expenses that exceed typical household savings (Kitaw et al. 2025). A high reimbursement limit establishes a financial safety threshold, which helps mitigate the probability of catastrophic expenditures. This preference is consistent with the loss aversion principle of prospect theory (Tversky and Kahneman 1991), where individuals place greater weight on avoiding losses than on acquiring equivalent gains.

In line with the MXL results in Table 3, the expansion of reimbursement coverage was the third most influential attribute. Current coverage under China's social health insurance is largely limited to the National Reimbursement Drug List (NRDL) and basic services (Jakovljevic et al. 2023). Significant gaps remain for high-cost diagnostics, specialized medical consumables, and innovative therapies. Our DCE results indicated a significant public preference for expanding this coverage, which may point to unmet needs for more comprehensive financial protection. In response to these gaps, policy reforms have been introduced since 2019, such as the National Healthcare Security Administration's centralized procurement program for high-cost medical devices (Central People's Government of the People's Republic of China 2025). For example, the inclusion of cochlear implants in the NRDL in 2021 reduced average patient out-of-pocket costs by ∼82%, significantly lessening the financial burden for affected families (Yi 2025).

However, interpreting these strong preferences for high reimbursement ratios and expanded coverage purely at face value overlooks critical systemic trade-offs regarding fund sustainability and equity. From a fiscal constraint perspective, expanding coverage depth to match residents’ ideal preferences could trigger substantial moral hazard and supplier-induced demand, threatening the long-term solvency of the public insurance fund. Furthermore, this creates a dilemma of resource prioritization across different socioeconomic groups. Since our sample overrepresents individuals with above-average incomes, their highlighted preference for comprehensive protection—especially the inclusion of high-cost, nonreimbursable services—might disproportionately benefit wealthier subgroups who can afford the residual out-of-pocket costs. For vulnerable or low-income populations (Oeben et al. 2025) (such as the “Reimbursement-Focused” Class 2 identified in our LCA), an uncritical expansion of benefit packages without structured premium subsidies could inadvertently lead to a regressive redistribution of public health financing resources.

### Comparison with international evidence on cost-sharing sensitivity

As shown in Table 3, the attributes for cost, deductible, and designated hospitals were not statistically significant. This may be explained by several factors. The sample had a relatively high proportion of respondents (70%) with above-average incomes. For this group, the premium difference between ¥440 and ¥530 may have been below a perceptible threshold, which may have limited price sensitivity (Fu et al. 2024). Similarly, the deductible range of ¥10 000–¥20 000 may have been too narrow to generate statistically detectable differences in preference (Tversky and Kahneman 1991). Alternatively, given the high expected costs of treating a critical illness, this deductible range may represent a relatively small portion of total expenses and thus had limited influence on decisions. When respondents prioritized coverage for catastrophic medical expenses, variations in the deductible may have been overshadowed by more influential attributes like the reimbursement ratio and limit. This aligns with behavioral economics principles, where the demand for risk mitigation can dominate cost-sharing considerations.

A second explanation relates to the experimental design parameters. The tested premium range (¥440–¥530) represented a narrow gradient, ∼0.8%–1.2% of Shandong's per capita disposable income. This limited variation may have been insufficient to elicit statistically significant preferences. Furthermore, the designated hospital attribute was presented as a binary (yes/no) choice. Some respondents may have misinterpreted “non-designated hospitals” as being completely ineligible for any reimbursement, rather than potentially receiving a lower reimbursement rate. This may have obscured the perceived utility of this attribute due to framing effects (Bridges et al. 2011).

Notably, this observed limited sensitivity to deductibles and premiums diverges from typical international experience in health insurance DCEs. In many voluntary commercial insurance markets or multipayer systems globally, cost-sharing mechanisms like premiums and deductibles act as dominant economic barriers that heavily suppress plan selection. For instance, DCE studies of standalone microinsurance in rural Malawi and Indonesia identify deductibles as the most decisive attribute for households with limited liquidity, while Iranian supplementary insurance participants explicitly trade off reimbursement rates against out-of-pocket thresholds (Abiiro et al. 2014, Kazemi Karyani et al. 2019, Titaley et al. 2025). By contrast, our respondents barely weigh deductibles when choosing critical illness plans. In our study context, however, because the primary risk being insured against is catastrophic illness involving astronomical costs, the financial threshold of a minor deductible variance is psychologically minimized by consumers, shifting the decision-making calculus from basic cost containment to absolute risk insulation.

### The shaper role of China’s universal healthcare context

The broader policy context may also help explain these results. Within China's universal basic medical insurance system, CII is often viewed as a supplementary layer of protection (Feng 2025), which may lead respondents to prioritize the intensity of coverage (e.g. reimbursement ratio) over other policy features. This observed preference for “coverage depth” aligns with theories in behavioral insurance economics, which suggest that consumers place particular weight on protection against extreme risks when making insurance decisions within a system that already provides basic coverage.

This institutional interplay clarifies how a near-universal primary health insurance system actively redirects and sharpens public preferences toward supplementary protection. Because China’s basic medical insurance schemes (URRBMI and UEBMI) already absorb the financial shocks of routine and low-cost healthcare utilization, residents are relieved from weighing day-to-day healthcare access costs. Consequently, their expectations for the CII scheme are uniquely institutionalized to target residual, tail-end risks—specifically, the high-cost diagnostics and innovative therapies currently excluded from the national registry. This structural arrangement offers a vital lesson for a broader policy audience: the baseline maturity and design of a nation's primary healthcare safety net will fundamentally shape how supplementary schemes are perceived, shifting the public's focus from horizontal coverage expansion to vertical catastrophic risk mitigation.

As presented in Table 4, the LCA confirmed heterogeneity in preferences, identifying two distinct subgroups: a Coverage-Intensity Sensitive Group and a Reimbursement-Focused Group. The first group, which tended to be older, employed, and married, showed relatively higher preferences for high reimbursement limits and comprehensive coverage. This pattern may align with life-cycle risk management strategies. In contrast, the second group consisted of younger respondents with more diverse economic backgrounds. Their preferences were mainly driven by reimbursement ratio attributes, with no statistically significant preference difference observed for deductible levels. These two distinct preference subgroups further reinforce our core trade-off argument: uniform generous benefit design fails to reconcile heterogeneous resident demands with constrained fiscal budgets, which reinforces the necessity of tiered insurance packages proposed in policy implications.

### Policy implications

Synthesizing our within-country trade-off analysis, cross-national DCE comparisons, and the institutional features of China’s two-tier health financing system, we put forward balanced policy recommendations that reconcile public preference demand with long-term fund sustainability. Policymakers may prioritize increasing the reimbursement ratio within sustainable fiscal constraints, potentially through tiered reimbursement schemes or higher ratios for specific critical illnesses and low-income groups. Concurrently, policymakers may moderately raise annual reimbursement caps, or even eliminate limits for vulnerable groups and patients with highly prevalent severe illnesses to strengthen financial protection. The expansion of the reimbursement scope should strategically include clinically necessary but high-cost items, such as special drugs and high-value medical consumables. Given the nonsignificant preference for the deductible attribute, maintaining it within the range of ¥10 000–20 000 may be reasonable, while premiums may be maintained or finely adjusted within the tested range, supplemented by targeted subsidies for low-income groups. To ensure long-term sustainability, establishing a dynamic adjustment mechanism for regular review of premiums, benefits, and fund solvency may be beneficial. Finally, strengthening policy communication and public education may help improve residents’ understanding and satisfaction with the insurance scheme.

### Limitations

This study has several limitations that should be acknowledged. First, the sample overrepresented younger and middle-to-high income residents of Shandong Province, and may not fully reflect the preferences of elderly, retired, low-income or unemployed groups. Second, the attribute levels for some design features, such as premium and deductible, covered only a relatively narrow range, which may have restricted the ability to detect more pronounced preference differences. Third, data on residents’ participation in other supplementary health insurance schemes were not collected, which may further moderate preferences for CII design.

In addition, the study was limited to respondents from Shandong Province, restricting the generalizability of the findings to other regional contexts. Future multiregional surveys would help verify broader applicability. As with most DCE studies, this research is subject to hypothetical bias, whereby stated preferences may not perfectly correspond to real-world behavioral decisions. The DCE employed a forced-choice design without an opt-out option, which means simulated choice probabilities reflect only relative preferences rather than actual uptake or enrollment behavior. The cross-sectional design also captures preferences only at a single time point and cannot account for dynamic changes over time, limiting causal interpretation. Furthermore, while attribute selection was based on prior qualitative work, other unmeasured contextual factors may still have influenced respondents’ hypothetical choices. Future research could combine DCE with follow-up surveys or real-world insurance data to mitigate these shortcomings.

## Conclusion

This study employed a DCE to explore resident preferences regarding CII scheme design in Shandong Province. The analysis identified three key attributes influencing preferences: reimbursement ratio, reimbursement limit, and expansion of coverage. The findings suggest that residents place priority on alleviating the heavy financial burden caused by catastrophic medical expenditure. Therefore, optimizing coverage provisions—particularly the reimbursement ratio, reimbursement limit, and coverage scope—may serve as a core direction to improve the rational design and public acceptance of CII arrangements. To help mitigate the risk of medical impoverishment and enhance equity in financial protection, policymakers may draw on these findings to inform refined resource allocation. Such efforts could contribute to developing a more targeted, equitable, and sustainable CII framework.

## Supplementary Material

czag089_Supplementary_Data

## Data Availability

The datasets generated and analyzed during the current study are not publicly available due to patient privacy concerns but are available from the corresponding author on reasonable request.

## Reflexivity statement

The author group of this paper comprises four females and two males, spanning early-career researchers to senior investigators. Our team includes members based in China (Shandong, Beijing, Zhejiang) and the Netherlands, reflecting both local and international perspectives on health policy and insurance research. Disciplinary backgrounds include public health, nursing, evidence-based medicine, health policy, and clinical medicine. All authors have substantial experience conducting quantitative and qualitative research in Chinese health settings, particularly in Shandong Province. We are committed to equitable research partnerships and to ensuring that our policy recommendations are grounded in the preferences of the populations they are intended to serve.

## References

[czag089-B1] Abiiro GA, Leppert G, Mbera GB et al Developing attributes and attribute-levels for a discrete choice experiment on micro health insurance in rural Malawi. BMC Health Serv Res 2014;14:. 10.1186/1472-6963-14-235 PMC4032866 24884920

[czag089-B2] Bridges JF, Hauber AB, Marshall D et al Conjoint analysis applications in health—a checklist: a report of the ISPOR Good Research Practices for Conjoint Analysis Task Force. Value Health 2011;14:403–13. 10.1016/j.jval.2010.11.013 21669364

[czag089-B3] Central People's Government of the People's Republic of China . *Opinion of the National Healthcare Security Administration on Supporting Measures for the Pilot Program of Centralized Drug Procurement and Use Organized by the State* [EB/OL] (2019-2-28). https://www.gov.cn/zhengce/zhengceku/2019-10/12/content_5438741.htm (6 May 2025, date last accessed)

[czag089-B4] Central People's Government of the People's Republic of China . *Guiding Opinions of Six Departments on Implementing Major Disease Insurance for Urban and Rural Residents* [EB/OL] (2012-8-24). https://www.gov.cn/gzdt/2012-08/31/content_2214223.htm (6 May 2026, date last accessed)

[czag089-B5] Chakrabarti A, Shankar A. Determinants of health insurance penetration in India: an empirical analysis. Oxf Dev Stud 2015;43:379–401. 10.1080/13600818.2015.1057116

[czag089-B6] Chen Q, Dong Y, Lyu X. Incorporating discrete choice experiments into long-term care insurance policy decisions: evidence from China. Front Public Health 2025;13:1511001. 10.3389/fpubh.2025.1511001 PMC11876386 40041181

[czag089-B7] Clark MD, Determann D, Petrou S et al Discrete choice experiments in health economics: a review of the literature. Pharmacoeconomics 2014;32:883–902. 10.1007/s40273-014-0170-x 25005924

[czag089-B8] de Bekker-Grob EW, Donkers B, Jonker MF et al Sample size requirements for discrete-choice experiments in healthcare: a practical guide. Patient. 2015;8:373–84. 10.1007/s40271-015-0118-z PMC4575371 25726010

[czag089-B9] Ding J, Yang Y, He M. Analysis of the cumulative effect of medical insurance integration and critical illness insurance on rural poor and vulnerable populations. Financ Theory Pract 2025;46:22–9. 10.16339/j.cnki.hdxbcjb.2025.06.003

[czag089-B10] Duan P, Wang J, Zhang R et al Quantitative analysis of the diffusion characteristics of China's long-term care insurance policy based on the PMC index model. Front Public Health 2025;13:1467062. 10.3389/fpubh.2025.1467062 PMC12137344 40475207

[czag089-B11] Einav L, Finkelstein A, Levin J. Beyond testing: empirical models of insurance markets. Annu Rev Econom 2010;2:311–36. 10.1146/annurev.economics.050708.143254 PMC3092551 21572939

[czag089-B12] Feng Y . Optimization strategies for the participation of commercial insurance in the cooperative model for major disease insurance for urban and rural residents. Hebei Enterprise 2025;1:107–110. 10.19885/j.cnki.hbqy.2025.01.025

[czag089-B13] Fu L, Han J, Xu K et al Incentivizing primary care utilization in China: the impact of health insurance coverage on health-seeking behaviour. Health Promot Int 2024;39:daae115. 10.1093/heapro/daae115 39243132

[czag089-B14] Fu W . Research on the Poverty Reduction and Prevention Effects of the Major Disease Insurance for Urban and Rural Residents in Shandong Province. Shandong University, 2022. 10.27272/d.cnki.gshdu.2022.004337.

[czag089-B15] GBD 2019 Human Resources for Health Collaborators . Measuring the availability of human resources for health and its relationship to universal health coverage for 204 countries and territories from 1990 to 2019: a systematic analysis for the Global Burden of Disease Study 2019. Lancet 2022;399:2129–54. 10.1016/S0140-6736(22)00532-3 PMC9168805 35617980

[czag089-B16] Gething K, Erku D, Scuffham P. DCE attribute development investigating public policy for the provision of medicinal cannabis. J Med Econ 2024;27:1232–44. 10.1080/13696998.2024.2405288 39297447

[czag089-B17] Global Burden of Disease Health Financing Collaborator Network . Evolution and patterns of global health financing 1995–2014: development assistance for health, and government, prepaid private, and out-of-pocket health spending in 184 countries. Lancet 2017;389:1981–2004. 10.1016/S0140-6736(17)30874-7 PMC5440770 28433256

[czag089-B18] Global Burden of Disease Health Financing Collaborator Network . Trends in future health financing and coverage: future health spending and universal health coverage in 188 countries, 2016–40. Lancet 2018;391:1783–98. 10.1016/S0140-6736(18)30697-4 PMC5946843 29678341

[czag089-B19] Gruber J, Levy H. The evolution of medical spending risk. J Econ Perspect 2009;23:25–48. 10.1257/jep.23.4.25 19946978

[czag089-B20] Jakovljevic M, Chang H, Pan J et al Successes and challenges of China's health care reform: a four-decade perspective spanning 1985–2023. Cost Eff Resour Alloc 2023;21:59. 10.1186/s12962-023-00461-9 PMC10469830 37649062

[czag089-B21] Jiang J, Chen S, Xin Y et al Does the critical illness insurance reduce patients’ financial burden and benefit the poor more: a comprehensive evaluation in rural area of China. J Med Econ 2019;22:455–63. 10.1080/13696998.2019.1581620 30744446

[czag089-B22] Joglekar R . Can Insurance Reduce Catastrophic Out-of-Pocket Health Expenditure? Mumbai, India: Indira Gandhi Institute of Development Research, 2008.

[czag089-B23] Kahneman D, Tversky A. Prospect theory: an analysis of decision under risk. Econometrica 1979;47:263–91. 10.2307/1914185

[czag089-B24] Kazemi Karyani A, Akbari Sari A, Woldemichael A. Eliciting preferences for health insurance in Iran using discrete choice experiment analysis. Int J Health Policy Manag 2019;8:488–97. 10.15171/ijhpm.2019.29 PMC6706965 31441289

[czag089-B25] Kitaw TA, Tilahun BD, Zemariam AB et al The financial toxicity of cancer: unveiling global burden and risk factors—a systematic review and meta-analysis. BMJ Glob Health 2025;10:e017133. 10.1136/bmjgh-2024-017133 PMC11815433 39929536

[czag089-B26] Li H, Chen Z, Huangfu H et al A study on the impact of critical illness insurance on residents’ catastrophic medical expenses. Health Econ Res 2025;42:28–32. 10.14055/j.cnki.33-1056/f.2025.03.009

[czag089-B27] Liu M, Zhang Q, Lu M et al Rural and urban disparity in health services utilization in China. Med Care 2007;45:767–74. 10.1097/MLR.0b013e3180618b9a 17667311

[czag089-B28] Ma H, Jia E, Ma H et al Preferences for public long-term care insurance among middle-aged and elderly residents: a discrete choice experiment in Hubei Province, China. Front Public Health 2023;11:1050407. 10.3389/fpubh.2023.1050407 PMC9909219 36778541

[czag089-B29] National Healthcare Security Administration . *Notice of the State Administration of Taxation on Implementing the Basic Medical Insurance Program for Urban and Rural Residents in 2022* [EB/OL] (2022-7-8). https://www.nhsa.gov.cn/art/2022/7/8/art_104_8398.html (6 May 2026, date last accessed)

[czag089-B30] Odahowski CL, Zahnd WE, Zgodic A et al Financial hardship among rural cancer survivors: an analysis of the medical expenditure panel survey. Prev Med 2019;129s:105881. 10.1016/j.ypmed.2019.105881 PMC7190004 31727380

[czag089-B31] Oeben M, Krewer A, Noack M. Consequences of healthcare coaching for long-term unemployed individuals with health restrictions. Health Psychol Res 2025;13:e81240018. 10.14440/hpr.0121

[czag089-B32] Prosser LA . Statistical methods for the analysis of discrete-choice experiments: a report of the ISPOR conjoint analysis good research practices task force. Value Health 2016;19:298–9. 10.1016/j.jval.2016.05.002 27325320

[czag089-B33] Rahmani H, Talebianpour H, Sharafi SE et al Development of attributes and levels of mental health insurance services using a discrete choice experiment. J Educ Health Promot 2023;12:134. 10.4103/jehp.jehp_433_22 PMC10312408 37397093

[czag089-B34] Richard P, Walker R, Alexandre P. The burden of out of pocket costs and medical debt faced by households with chronic health conditions in the United States. PLoS One 2018;13:e0199598. 10.1371/journal.pone.0199598 PMC6016939 29940025

[czag089-B35] Shandong Provincial People's Government . *Opinions on Implementing the Major Disease Insurance Under the New Rural Cooperative Medical Scheme (Trial)* [EB/OL] (2012-10-16). http://www.shandong.gov.cn/art/2012/10/17/art_100623_28699.html (6 May 2025, date last accessed)

[czag089-B36] Soekhai V, De Bekker-Grob EW, Ellis AR et al Discrete choice experiments in health economics: past, present and future. Pharmacoeconomics 2019;37:201–26. 10.1007/s40273-018-0734-2 PMC6386055 30392040

[czag089-B37] Titaley CR, Ariawan I, Wahyuningsih W et al Use of the National Health Insurance among beneficiaries in Maluku province, Indonesia: a cross-sectional analysis of the 2021 social health insurance sample data. BMJ Open 2025;15:e106013. 10.1136/bmjopen-2025-106013 PMC12625941 41248413

[czag089-B38] Tversky A, Kahneman D. Loss aversion in riskless choice: a reference-dependent model*. Q J Econ 1991;106:1039–61. 10.2307/2937956

[czag089-B39] Vrieze SI . Model selection and psychological theory: a discussion of the differences between the Akaike information criterion (AIC) and the Bayesian information criterion (BIC). Psychol Methods 2012;17:228–43. 10.1037/a0027127 PMC3366160 22309957

[czag089-B40] Wagstaff A, Flores G, Hsu J et al Progress on catastrophic health spending in 133 countries: a retrospective observational study. Lancet Glob Health 2018;6:e169–79. 10.1016/S2214-109X(17)30429-1 29248367

[czag089-B41] Wagstaff A, Van Doorslaer E. Catastrophe and impoverishment in paying for health care: with applications to Vietnam 1993–1998. Health Econ 2003;12:921–34. 10.1002/hec.776 14601155

[czag089-B42] Xiao L, Min H, Wu Y et al Public's preferences for health science popularization short videos in China: a discrete choice experiment. Front Public Health 2023;11:1160629. 10.3389/fpubh.2023.1160629 PMC10436607 37601206

[czag089-B43] Xie X, Hu Y. The reimbursement rate of new rural cooperative medical scheme and self-rated health among rural middle-aged and elderly. Front Public Health 2022;10:627169. 10.3389/fpubh.2022.627169 PMC9024035 35462832

[czag089-B44] Yan N, Liu T, Xu Y et al Healthcare preferences of the general Chinese population in the hierarchical medical system: a discrete choice experiment. Front Public Health 2022;10:1044550. 10.3389/fpubh.2022.1044550 PMC9713319 36466449

[czag089-B45] Yi S . More Hearing-Impaired Individuals Are Using Cochlear Implants. People’s Daily 2025:15. 10.28655/n.cnki.nrmrb.2025.004894

[czag089-B46] Zhai S, Yuan S, Dong Q. The impact of health insurance on poverty among rural older adults: an evidence from nine counties of western China. Int J Equity Health 2021;20:47. 10.1186/s12939-021-01379-5 PMC7831180 33494750

[czag089-B47] Zhang J, Li Q, Zhang J et al Chinese university students’ preferences for physical activity incentive programs: a discrete choice experiment. Front Public Health 2023;11:1281740. 10.3389/fpubh.2023.1281740 PMC10646335 38026342

[czag089-B48] Zhou Y, Wushouer H, Vuillermin D et al Does the universal medical insurance system reduce catastrophic health expenditure among middle-aged and elderly households in China? A longitudinal analysis. Eur J Health Econ 2021;22:463–71. 10.1007/s10198-021-01267-3 33582893

