# Supplementary material for: Residents’ preference trade-offs for critical illness insurance scheme design: a discrete choice experiment in Shandong Province, China
Source: Health Policy Plan. 2026 Jul 16;41(8):1388–400. doi: 10.1093/heapol/czag089 (PMC13586614; doi:10.1093/heapol/czag089)
Supplement: Supplementary file 1 [file czag089_supplementary_data.docx]

**Supplementary**

Table 1 Estimates of the conditional logit model

| attribute | *β* | *se* | *p* | 95%CI |
| --- | --- | --- | --- | --- |
| Cost | 0.001 | 0.002 | 0.469 | (-0.003-0.005) |
| Reimbursement ratio(reference 60%) | | | | |
| 65% | -0.688 | 0.229 | 0.003 | (-1.136--0.240) |
| 70% | 0.475 | 0.200 | 0.018 | (0.083-0.867) |
| 75% | 1.002 | 0.076 | 0.000 | (0.854-1.150) |
| Deductible(reference ¥10000) | | | | |
| ¥15000 | -0.170 | 0.169 | 0.313 | (-0.501-0.161) |
| ¥20000 | -0.545 | 0.505 | 0.281 | (-1.534-0.445) |
| Annual reimbursement limits(reference ¥400,000) | | | | |
| ¥500,000 | -0.313 | 0.148 | 0.034 | (-0.603--0.023) |
| ¥600,000 | 0.709 | 0.280 | 0.011 | (0.160-1.259) |
| boundless | 0.964 | 0.369 | 0.009 | (0.242-1.687) |
| Expansion of reimbursement coverage(reference Special medical services in the top 10% of out-of-catalogue expenses) | | | | |
| reference Special medical services in the top 20% of out-of-catalogue expenses | 0.488 | 0.132 | 0.000 | (0.229-0.746) |
| reference Special medical services in the top 30% of out-of-catalogue expenses | 1.155 | 0.204 | 0.000 | (0.756-1.554) |
| Designated hospitals(reference Yes) | | | | |
| No | 0.273 | 0.259 | 0.292 | (-0.234-0.779) |
| Log likelihood | -2424.189 |  |  |  |
| AIC | 4872.378 |  |  |  |
| BIC | 4956.249 |  |  |  |
| Observations | 8014 |  |  |  |
| Sample size | 334 |  |  |  |
